# Aging the brain: multi-region methylation principal component based clock in the context of Alzheimer’s disease

**DOI:** 10.18632/aging.204196

**Published:** 2022-07-30

**Authors:** Kyra L. Thrush, David A. Bennett, Christopher Gaiteri, Steve Horvath, Christopher H. van Dyck, Albert T. Higgins-Chen, Morgan E. Levine

**Affiliations:** 1 Program in Computational Biology and Bioinformatics, Yale University, New Haven, CT 06511, USA; 2 Rush Alzheimer’s Disease Center, Rush University Medical Center, Chicago, IL 60612, USA; 3 Department of Human Genetics, David Geffen School of Medicine, UCLA, Los Angeles, CA 90095, USA; 4 Department of Biostatistics, Fielding School of Public Health, UCLA, Los Angeles, CA 90095, USA; 5 Department of Psychiatry, Yale University School of Medicine, New Haven, CT 06511, USA; 6 Alzheimer’s Disease Research Center, Yale University School of Medicine, New Haven, CT 06510, USA; 7 VA Connecticut Healthcare System, West Haven, CT 06516, USA; 8 Department of Pathology, Yale University School of Medicine, New Haven, CT 06519, USA; 9 Altos Labs, San Diego Institute of Science, San Diego, CA 92114, USA

**Keywords:** epigenetic clocks, unsupervised machine learning, brain, Alzheimer's disease, age acceleration

## Abstract

Alzheimer’s disease (AD) risk increases exponentially with age and is associated with multiple molecular hallmarks of aging, one of which is epigenetic alterations. Epigenetic age predictors based on 5’ cytosine methylation (DNAm), or epigenetic clocks, have previously suggested that epigenetic age acceleration may occur in AD brain tissue. Epigenetic clocks are promising tools for the quantification of biological aging, yet we hypothesize that investigation of brain aging in AD will be assisted by the development of brain-specific epigenetic clocks. Therefore, we generated a novel age predictor termed PCBrainAge that was trained solely in cortical samples. This predictor utilizes a combination of principal components analysis and regularized regression, which reduces technical noise and greatly improves test-retest reliability. To characterize the scope of PCBrainAge’s utility, we generated DNAm data from multiple brain regions in a sample from the Religious Orders Study and Rush Memory and Aging Project. PCBrainAge captures meaningful heterogeneity of aging: Its acceleration demonstrates stronger associations with clinical AD dementia, pathologic AD, and APOE ε4 carrier status compared to extant epigenetic age predictors. It further does so across multiple cortical and subcortical regions. Overall, PCBrainAge’s increased reliability and specificity makes it a particularly promising tool for investigating heterogeneity in brain aging, as well as epigenetic alterations underlying AD risk and resilience.

## INTRODUCTION

Aging is the most significant and consistently demonstrated risk factor for Alzheimer’s disease (AD) [1–3]. As a result, the aging of the U.S. population is expected to coincide with a rise in AD cases, increasing from 5.8 million in 2020, to 13.8 million projected by 2050 [4]. Chronological age, defined as time since birth, is a non-modifiable risk factor. Biological aging however, or the molecular and cellular changes that underlie the process of aging, may be malleable [5–7]. Approaching the challenge of AD prevention and treatment through the lens of biological aging thus provides a major opportunity for improving cognitive health and reducing disease burden.

Because the brain is the central site for AD pathology, understanding the specific aging of this tissue is a priority. As brain tissue ages, misfolded tau and amyloid proteins accumulate due to a loss of proteostasis, one molecular hallmark of aging [8]. While this occurs in older adults with normal cognition or mild cognitive impairment, it is generally more pronounced in subjects with AD dementia [9–11]. Neuritic plaque and neurofibrillary tangles form the basis for the neuropathological diagnosis of AD [12]. Further, more rapid accumulation of tau [13] and β-amyloid [14] aggregates is also linked to inheritance of the APOE ε4 allele, which is itself linked to a number of age-related outcomes, including increased AD dementia risk [15], CVD risk [16, 17], and reduced lifespan [18, 19]. This suggests that while AD may not be a normal part of aging, it is partially driven by changes that are known to relate to basic aging processes.

Additional hallmarks of biological aging, which include epigenetic alterations [20, 21], have also been implicated in the pathology of AD. For instance, 5’ cytosine methylation (DNAm) differences have been shown to track aging and can be quantitatively combined to produce composite aging biomarkers, termed “epigenetic clocks” [22]. We and others have shown that the divergence between observed and predicted ages produced by epigenetic clocks relate to AD pathology. For instance, Horvath pan-tissue [23] and Levine PhenoAge [24] epigenetic age acceleration positively correlate with neuritic plaque, NFT, and β-amyloid loads. While this provides further molecular evidence of a link between AD risk and measurably accelerated biological aging, such clocks are typically developed in peripheral tissues and may not capture the unique aging changes in the brain. A notable exception is the recent DNAmClock_Cortical_ [25]. A recent study using Cortical tissue found that the DNAmClock_Cortical_ had much stronger associations with AD clinical and neuropathologic traits relative to Horvath, Hannum and PhenoAge clocks based on non-neuronal tissues [26]. Building on previous work, our study incorporates two additional novel features. First, we sought to investigate the extent to which the uneven pathological burden evident by amyloid [27] and tau [28] staging is captured when considering DNAm across multiple paired brain regions, rather than in singular areas like the cortex or hippocampus. Second, we have previously shown that CpG clocks often suffer from significant technical noise, hindering their applications. Therefore, we sought to use our recently developed approach to improve signal-to-noise ratios in methylation data, leading to improved reliability and construct validity in our novel epigenetic clock [29].

Overall, we hypothesized that a brain age methylation-based predictor could be developed with meaningful disease associations and broad multi-brain-region utility. To test this, we used DNAm capture to generate a PC-based epigenetic predictor of brain aging which we show to: (1) strongly reflect AD neuropathology and cognitive decline, and (2) track age across multiple brain regions. This resulting measure, PCBrainAge, is applicable for use in existing brain and tissue banks, and many publicly available postmortem datasets for the study of AD. It is available as an R package at https://github.com/MorganLevineLab/calcPCBrainAge.

## RESULTS

### Model design and testing

To generate a predictor of aging in the brain, we selected a publicly accessible dataset deposited into the Gene Expression Omnibus [30] (GSE74193) [31]. In brief, this dataset contains methylation data from dorsolateral prefrontal cortex (DLPFC) of 399 individuals aged 20+ (see Methods for further details). This dataset includes patients diagnosed with schizophrenia (*n* = 187; 47%). However, this neuropsychiatric disease has not been shown to be robustly associated with epigenetic signatures of chronological age in either blood or brain, despite acceleration in clocks predicting mortality in blood [32, 33]. Our model’s outcome variable is chronological age, so inclusion of schizophrenia samples is reasonable and potentially advantageous: Inclusion of schizophrenia samples reduces the likelihood that general brain pathology will exert a large impact on the model’s predictions, as the model is forced to predict chronological age despite schizophrenia status. Nevertheless, as a sensitivity analysis, we also trained a model using only control individuals, which did not improve results (Supplementary Figure 1). Thus, we included all high-quality samples for training, regardless of schizophrenia status.

The training method to generate our predictor is built upon our recently published PC Clock method [29]. In brief, singular vector decomposition (SVD), an extension of principal components analysis suitable for wide format data (i.e., where features outnumber samples), was performed on this training methylation dataset. This analysis was limited to CpG sites that are overlapped between the training, test, and validation datasets collected on 450K or EPIC arrays (see Methods for more detail). This produced 399 left singular vectors, which for general purposes, are referred to here as principal components (PCs) of 5′-cystosine methylation in postmortem dorsolateral prefrontal cortex (DLPFC). The PC scores, representing an individual’s projection values onto the principal component vectors, were used as the set of variables from which age was predicted via elastic net penalized regression.

**Figure 1:**
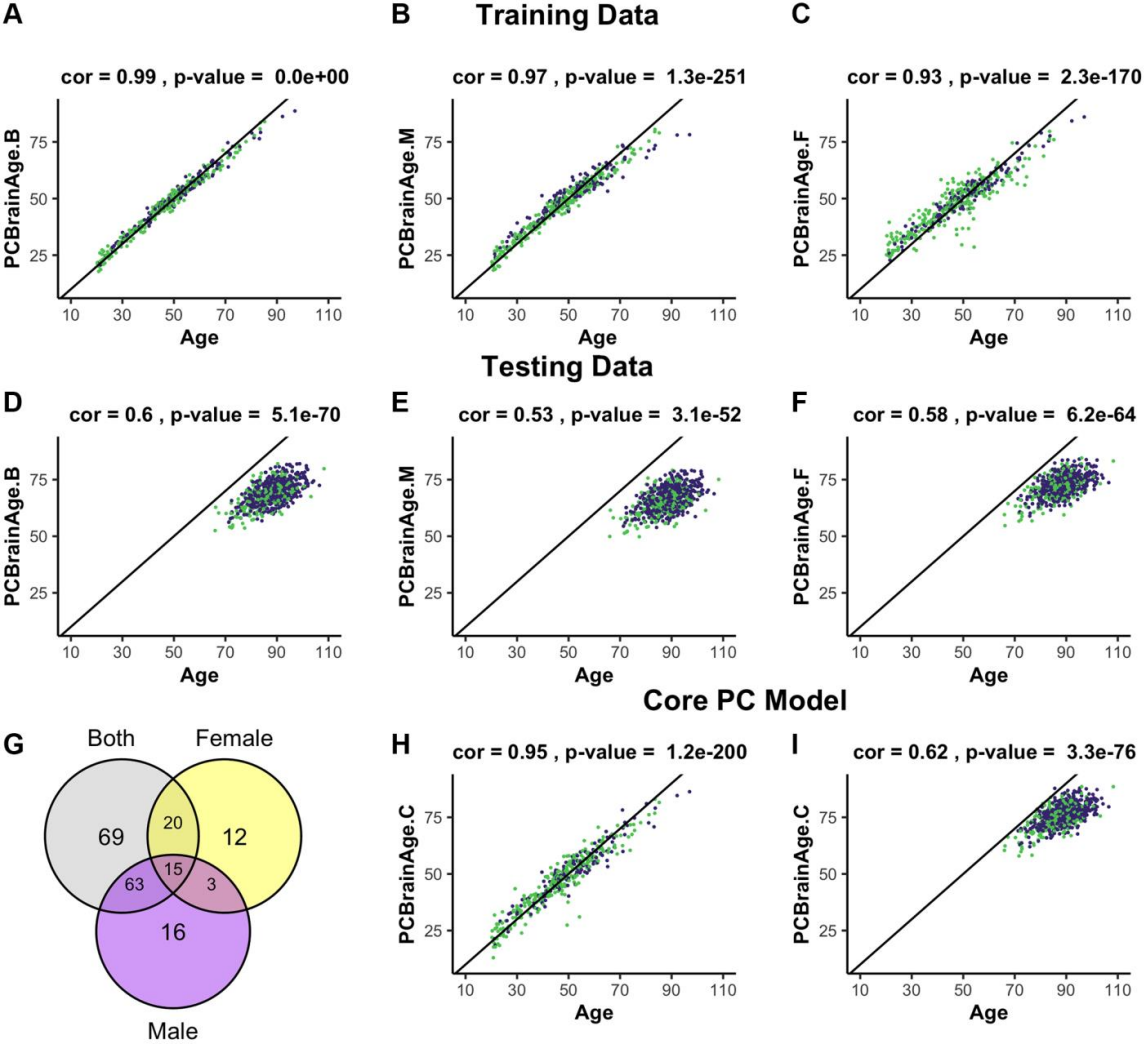
**Training and testing of multiple iterations of PCBrainAge.** Using the dataset from GSE74193, elastic net was used to predict age using principal component loadings in both sexes (**A**), only males (**B**), or only females (**C**). Here, we show the resultant predictions for each model in both females (purple) and males (green) regardless of training sex. Each model so trained is then predicted in all individuals from syn5850422 (**D**–**F**), regardless of sex or AD status. Each model selected a number of principal components to use for prediction, and we compared the selection of each model using a Venn diagram (**G**). Subsequent training of an elastic net model using only the 15 core principal components in both sexes is visualized (**H**) and compared to performance in the test dataset (**I**).

To predict training sample age, three models were generated, differing in the sex representation of subjects. This choice was based upon known sex-specific differences in aging [34], and evidence of sex-specific differences in AD risk and AD neurobiology [35]. All models used elastic net penalized regression in the appropriate individuals to find the optimal weighted linear average of PCs to predict chronological age; the first utilized both sexes (*n* = 399, Figure 1A, 1D); the second was fit to only males (*n* = 262, Figure 1B, 1E); the third was fit to only females (*n* = 137, Figure 1C, 1F). Regardless of the sample used for training, we found that each model attained similar correlation between predicted and chronological age in both males and females. Male- and female-specific age correlations for each model can be found in Table 1.

**Table 1:** Pearson’s correlation of age by sex with model age predictions.

**Model**		**Training data**	**Core**	**Test data**	**Core**
**Both Sexes**	M	0.99	0.94	0.61	0.65
	F	0.99	0.95	0.56	0.58
**Male**	M	0.98		0.55	
	F	0.95		0.49	
**Female**	M	0.90		0.61	
	F	0.97		0.54	

A total of 195 of 399 PCs were selected for use in one or more models, with the female model (PCBrainAge.F) selecting the fewest variables. However, a completely overlapped set of 15 PCs (referred to as the core PCs) were selected for all three models, representing an important centralized signal of aging (Figure 1G). The creation of three degenerate models in this manner allowed us to isolate a robust brain aging signal. We investigated whether these core PCs were significantly more important than their non-core counterparts in each model. To do so, we sequestered the 15 core PCs in the training data, as well as 3 different sets of non-core PCs corresponding to each original model. Using the same elastic net regression procedure as the original models, we regressed the core and noncore PC scores to age in the appropriate training subcohorts. The 15 core principal components were sufficient to predict age in the training data. However, the non-core models were unable to successfully do so (Supplementary Figure 2). Therefore, we generated a final model PCBrainAge.C. This was trained in both males and females and includes only the 15 core PCs (Figure 1H). As the core PC version of PCBrainAge is the overall superior model—both requiring limited information and performing best—only this model is used hereafter and will be simply referred to as PCBrainAge for clarity.

To validate models of aging generated from training data, an independent methylation dataset of 718 DLPFC samples was obtained through Synapse (syn5850422) [36]. All datasets used in the current work are characterized in Supplementary Table 1. Estimation of the individuals’ PC loadings was performed by projection onto the right singular vectors of the training dataset, thereby generating the 399 training PC vectors based upon the original eigenvalue estimations from the training data [29]. In terms of age predictions, PCBrainAge.C performs at least as well as all original sex-stratified and full models in the test dataset (Figure 1I), despite using fewer principal components.

**Figure 2:**
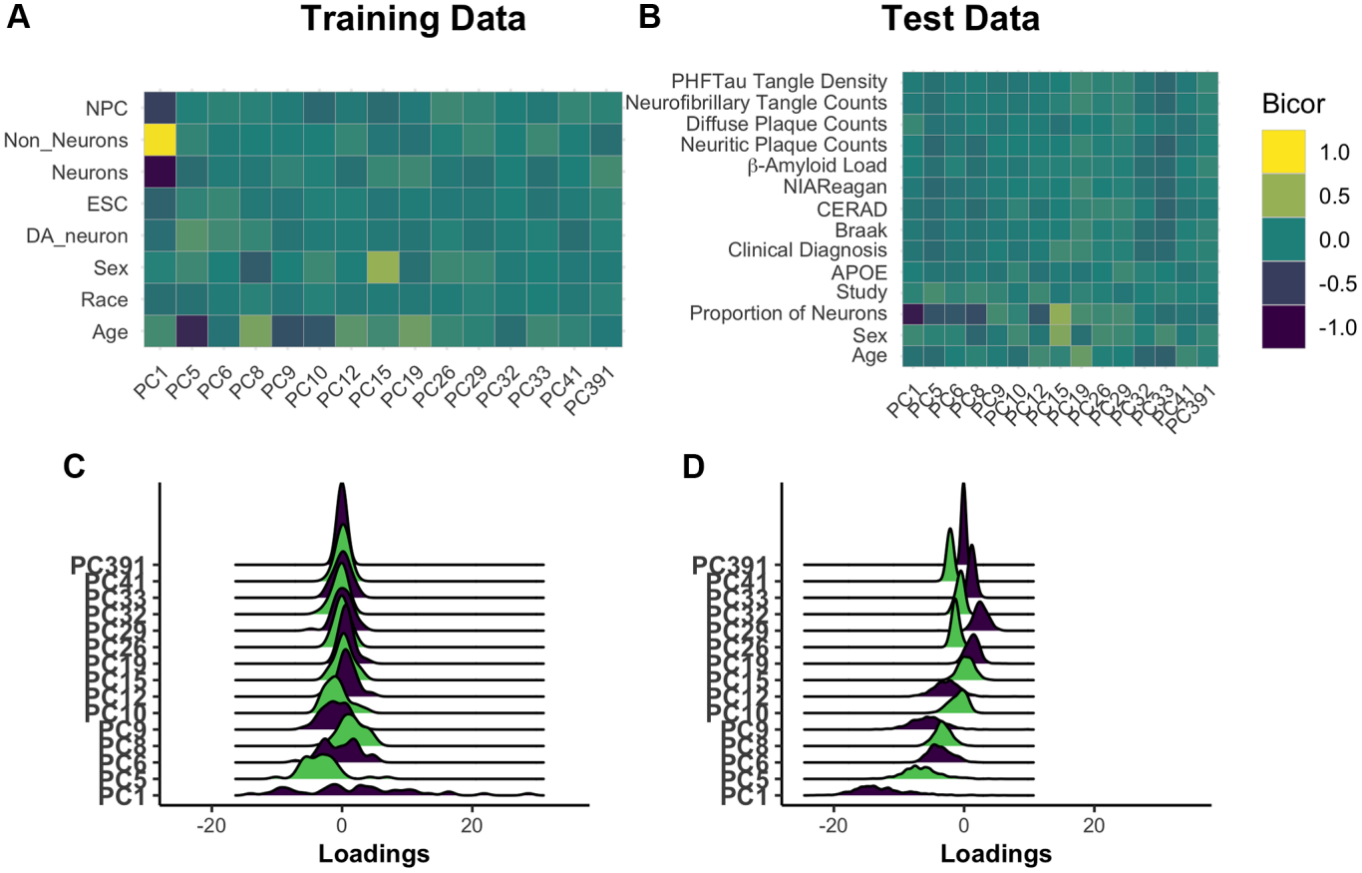
**Understanding core principal component composition.** Principal component loadings for individuals in the training dataset were correlated using biweight midcorrelation (bicor) to selected author-provided phenotypic annotations (**A**). The same procedure was applied to the projected principal component loadings for all individuals in the test dataset, including those with and without Alzheimer’s disease (**B**). To ensure that future correlations between age prediction and disease are not the result of unrealistic distortions in PC loadings following the prediction process, we used ridgeplots to visualize the distribution of loadings in each PC in age 65+ training individuals (**C**) and the test data (**D**). [Abbreviations: NPCs: neural progenitor cells; Cort: cortical; ESCs: embryonic stem cells; DA: dopaminergic].

Principal components are complex, composite variables, making them challenging to interpret. To investigate the information captured in the 15 core PCs, we correlated each PC to annotated features of the training dataset. This demonstrated that the largest source of variation in the data, as captured by PC1, is cell composition (Figure 2A). PC5 is most strongly correlated with age (|r| = 0.68), with all PCs having a range of absolute biweight midcorrelation of 0.04–0.68. As the overall model has correlation with age of 0.95, the signal for chronological age is clearly distributed across PCs. PC8 and PC15 are related to biological sex. These observations were confirmed when the PCs were projected onto the test data (Figure 2B, Supplementary Figure 3). While neuron proportion demonstrates correlations with more PCs in the test data compared to training data, there are several explanations for this observation. First, it is consistent with an expected, subtle loss of the imposed orthogonality when principal components are projected into new datasets. Second, our test dataset is comprised of only older adults, many with AD, who are expected to have age-associated neuron loss. This may enhance correlations between cell composition and methylation PCs that were not otherwise apparent in training data capturing the entire lifespan. Third, the proportion of neurons in test data, unlike training data, is estimated using the methylation itself. Therefore, this estimated cell proportion may be doubly affected by disease states or other signals being captured in the data. Finally, in the test dataset, where some individuals have AD and dementia, we find that no single PC is highly and/or consistently correlated to AD status (Supplementary Table 2).

We checked for relative agreement between training and testing data composition prior to applying PCBrainAge to test data. We previously showed that it is possible to use PCs from one dataset to generate reliable and useful PCs when projecting to another [29]. However, there can be differences in the distributions of PC scores: Therefore, we analyzed the distribution of PC scores from the core and found that while there are shifts in the mean, the general shape remains intact (Figure 2C, 2D). This can contribute to differences in the intercept in new datasets, a known behavior in common CpG clocks. However, this is easily corrected by using age acceleration, which does not consider intercept, for further analyses.

### PCBrainAge correlates with Alzheimer’s pathology in DLPFC

**Figure 3:**
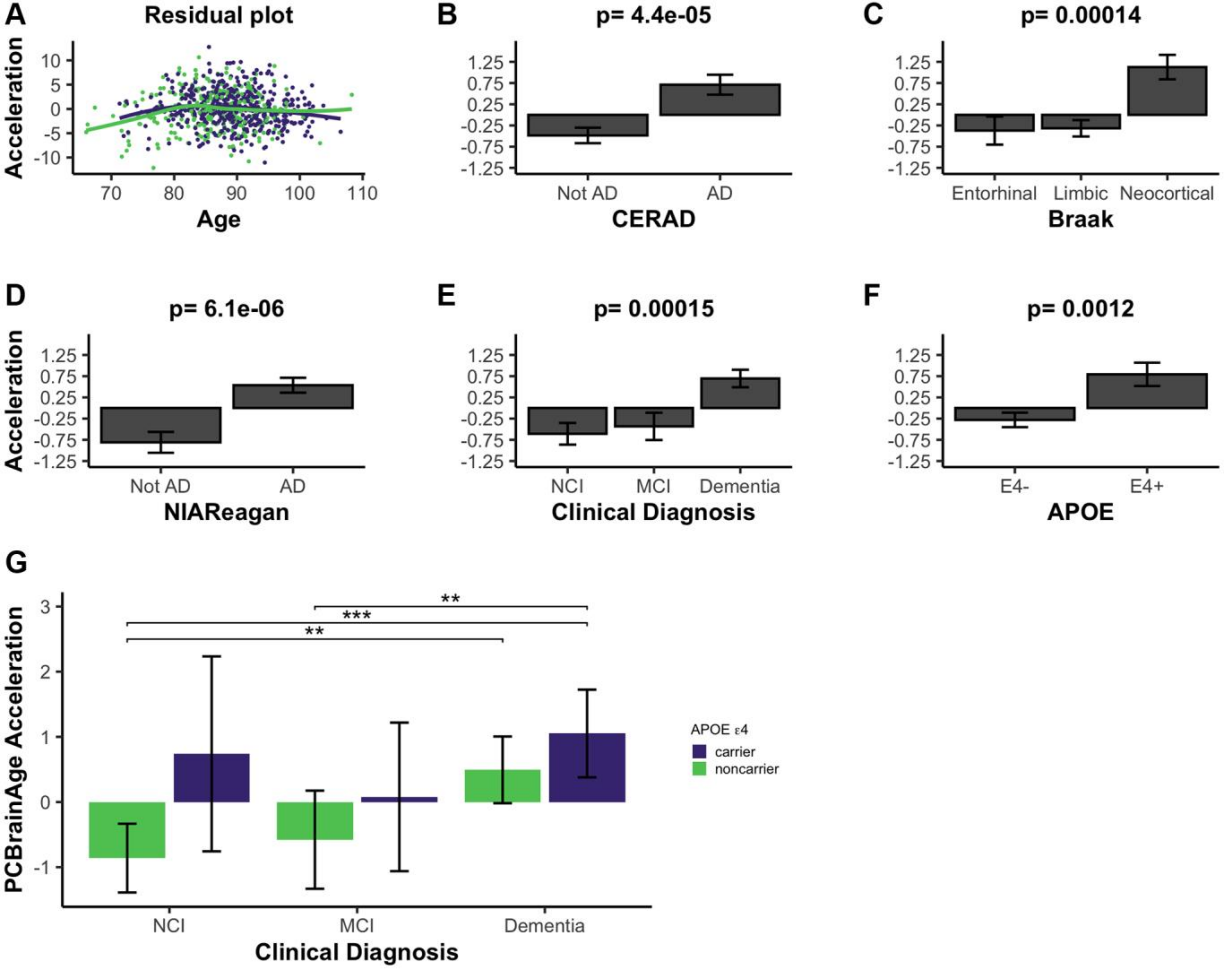
**PCBrainAge acceleration is associated with indications of AD.** (**A**) PCBrainAge residuals following multiple correction were verified to remain orthogonal to age using a scatterplot with LOESS curves for males (green) and females (purple). PCBrainAge Acceleration was subsequently analyzed in the context of CERAD scores (**B**), Braak stages (**C**), NIA Reagan scores (**D**), the ante-mortem clinical diagnosis (**E**), and the APOE ε4 carrier status (**F**) of each individual. *P*-values are the result of performing Kruskal-Wallis tests of nonparametric means amongst the categorical groups. Error bars for 3B-3F depict 1 standard error. (**G**) Acceleration was further broken down into cognitive groups by APOE ε4 carrier status for improved clarity. Error bars depict the 95% confidence interval. Significance levels based on BH adjusted *p* values are: ^*^P < 0.05, ^**^P < 0.01; ^***^P < 0.001.

We calculated brain age acceleration in the ROSMAP DLPFC test dataset (*n* = 700) by generating linear models to regress PCBrainAge on samples’ true ages at death and proportion of neurons (Supplementary Table 3). Proportion of neurons was explicitly included to obtain residuals, as we hypothesized that cell proportion changes appear to be the dominant signal in data (Figure 2A, 2B). Ultimately, we are interested in whether PCBrainAge is predictive of AD beyond the well-characterized impacts of changes to neuron abundance. This is also consistent with previous reports that accounting for cell type heterogeneity improves mortality and biological age prediction [37, 38]. Age acceleration was correlated with pathological and phenotypic traits known to indicate or affect the course of AD. To ensure that such correlations were not impacted by a nonlinear relationship of age and PCBrainAge, we verified a uniform distribution of residuals in both sexes (Figure 3A). Slight nonlinearity at the extremes of the distribution appeared to be the result of reduced sample density rather than true nonlinearity.

PCBrainAge accelerations were tested for association with AD clinical and pathologic diagnosis, and APOE ε4 status. Postmortem binarized AD diagnosis according to neuritic plaque derived CERAD scores is significantly associated with accelerated brain aging (Figure 3B), as well as neurofibrillary tangle (NFT) derived Braak Staging (Figure 3C). Notably, PCBrainAge is significantly accelerated when AD is in the neocortical, final stages versus all prior stages, but has no discriminatory power between the entorhinal and limbic phases. This likely reflects that PCBrainAge’s training and testing are occurring in DLPFC, a neocortical region. PCBrainAge acceleration is also positively associated with post-mortem neuropathologic AD diagnosis by combined neuritic plaque (np) and NFT to derive NIA Reagan score (Figure 3D), as well as ante-mortem clinical diagnosis of AD dementia (Figure 3E). Those with the clinical diagnosis of mild cognitive impairment (MCI) are indistinguishable from non-cognitively impaired individuals. Thus, individuals with AD show greater PCBrainAge acceleration than their counterparts. The APOE ε4 allele has been reproducibly associated with AD risk, and earlier onset of the disease [39]. Positive APOE ε4 status (i.e., carrying 1 or 2 APOE ε4 alleles) was significantly associated with PCBrainAge acceleration (Figure 3F). In fact, PCBrainAge is accelerated across APOE ε4 carriers such that cognitively normal and AD confirmed individuals are indistinguishable. In contrast, among non-carriers those with AD show significant acceleration over premortem cognitively normal individuals (Figure 3G).

**Figure 4:**
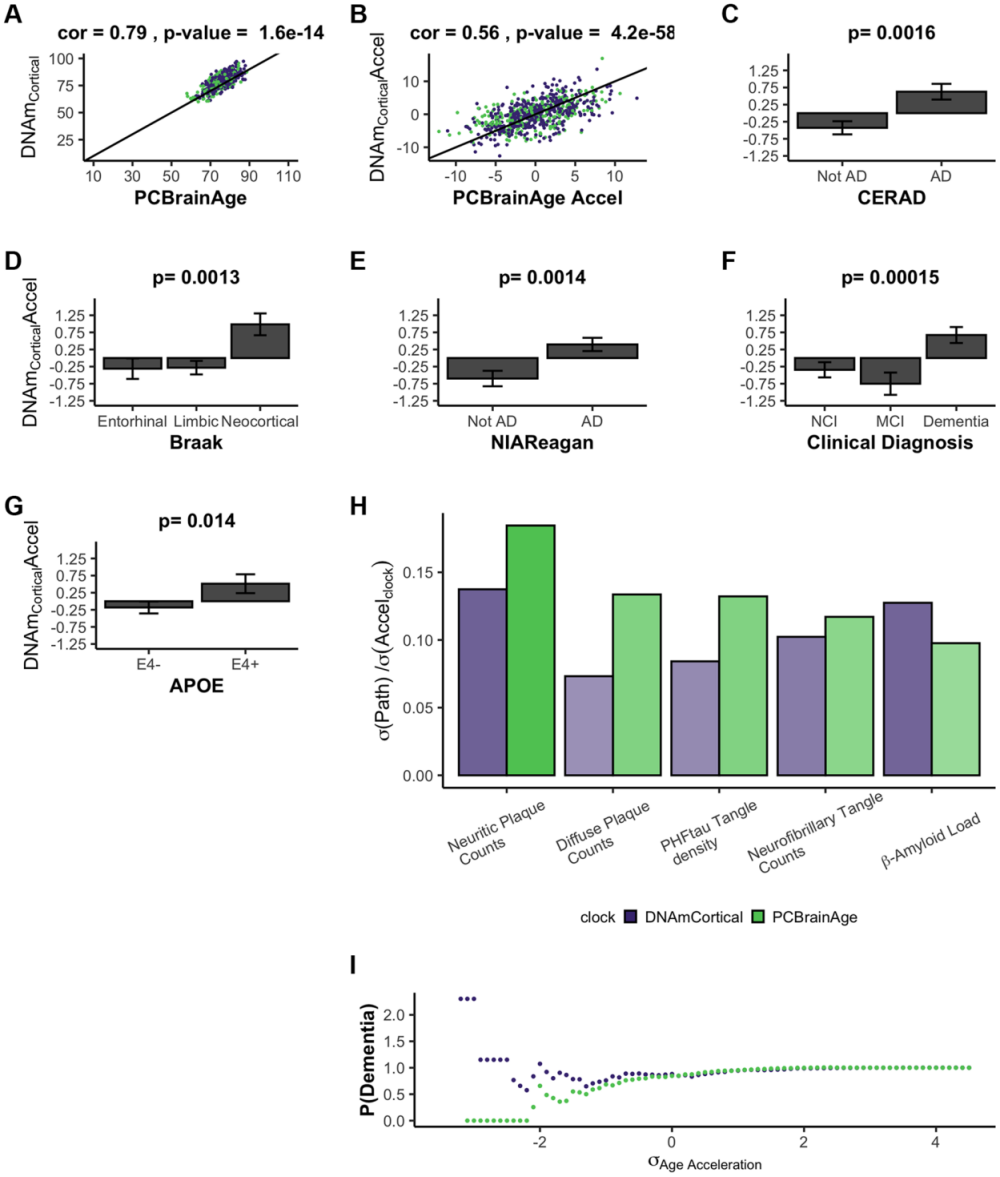
**DNAmClock_Cortical_ prediction in test data comparable to PCBrainAge predictions.** DNAmClock_Cortical_ was estimated in our test dataset, which is independent from its original training. We find that DNAmClock_Cortical_ has moderate correlation with age at death (**A**), and agreement with PCBrainAge accelerations for the same individuals (**B**). While DNAmClock_Cortical_ does exhibit clear acceleration in (advanced) AD patients (**C**–**E**), demented patients (**F**), and APOEε4 carriers (**G**), the *p*-values of the separation between groups are slightly attenuated versus those of PCBrainAge (see Figure 3). The standard deviation of various AD pathological characteristics per clock standard deviation are compared for DNAmCortical (pink) and PCBrainAge (blue) (**H**). Given individuals less than or equal to a standard deviation of age acceleration for each clock, the probability of patients being diagnosed with dementia normalized to the total cohort probability is shown for each clock (**I**).

During the course of the current research, another methylation-based epigenetic clock was reported, termed DNAmClock_Cortical_ [25]. As expected, DNAmClock_Cortical_ is correlated with PCBrainAge for both predicted age and age acceleration (r = 0.79 and r = 0.56, respectively) (Figure 4A, 4B). The training samples of DNAmClock_Cortical_ (*n* = 1,047) included the samples used to train PCBrainAge (*n* = 399) and was intended to accurately estimate chronological age of samples at all ages (training and test samples aged 1–108). Indeed, DNAmClock_Cortical_ does show better correlations with chronological age compared to PCBrainAge. However, our work with PCBrainAge intends to not only predict age, but to capture relevant biological heterogeneity of aging in the brain—especially that associated with AD. DNAmClock_Cortical_ acceleration shows less significant associations with AD clinical and pathologic phenotypes, and APOE ε4 carrier status in comparison with PCBrainAge (Figure 4C–4G compared to Figure 3). This then suggests that DNAmClock_Cortical_, in optimizing prediction of chronological age, may miss relevant heterogeneity and aging signals associated with AD. A 1-SD change in DNAmClock_Cortical_ acceleration does correspond to odds of pathologic AD, but this is mostly limited to amyloid and neuritic plaques. However, a 1-SD difference in PCBrainAge reflects greater differences with AD pathology, and is more balanced across various postmortem metrics of AD pathology (Figure 4H). Further, it was found that increasing standard deviations of PCBrainAge acceleration show monotonic increases in the normalized probability of dementia, unlike the stochasticity observed in DNAmClock_cortical_ (Figure 4I). This is likely reflective of the hypothesized reduction in noisy CpGs and improved resolution expected to arise from using our PC Clocks method.

The association of PCBrainAge acceleration and AD pathology suggests a discriminatory role for PCBrainAge beyond age prediction itself. Increased prediction accuracy of chronological age may reduce the association with AD. PCBrainAge provides meaningful, nonrandom information about both age and the disease status of the brain.

### PCBrainAge demonstrates improved test-retest reliability in brain data

**Figure 5:**
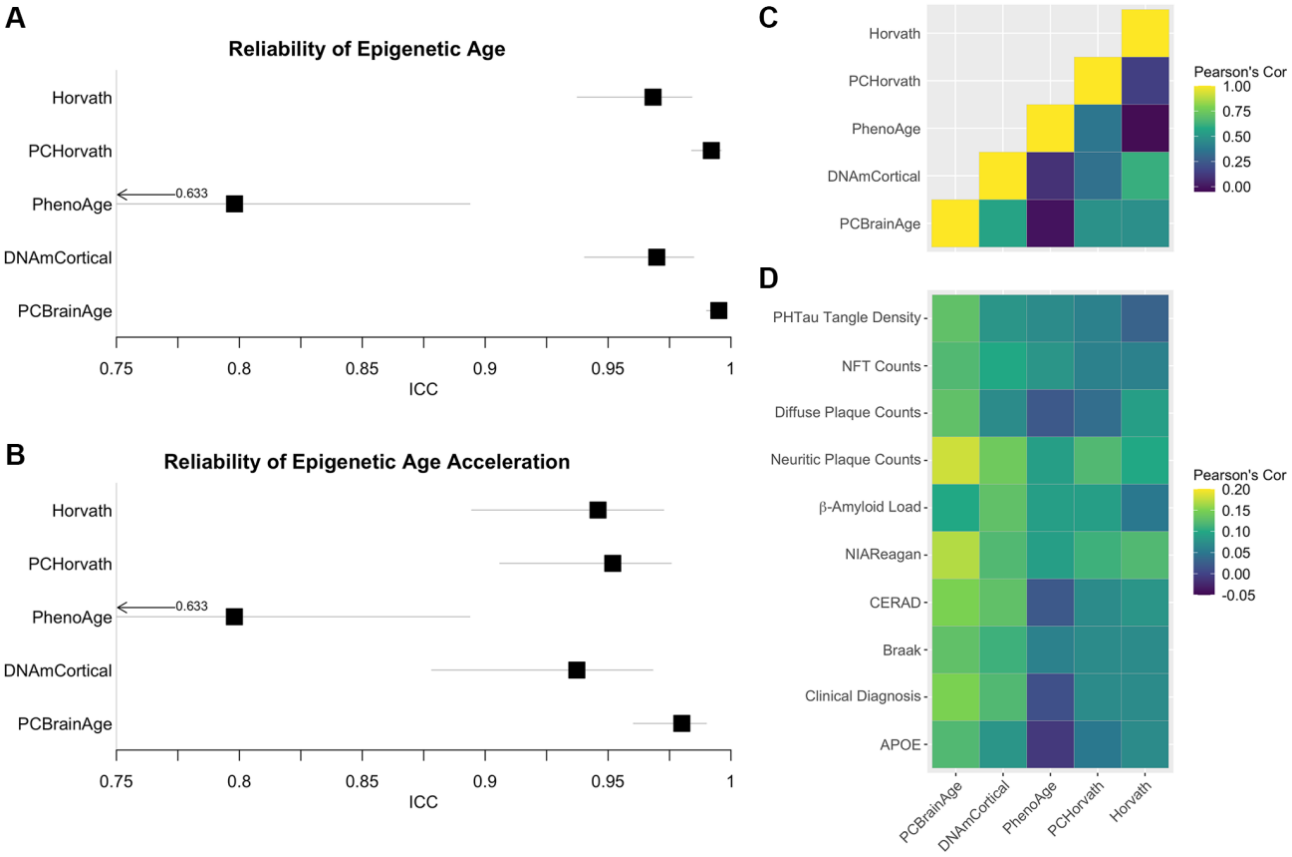
**Reliability of Alzheimer’s associated DNAm clocks and correlated pathology.** Test-retest reliability of DNAm clocks previously reported to associate with clinical or pathological criteria of AD was measured using two-way consistency ICC values, in a dataset of 34 pairs of cerebellum replicates (**A**). The procedure was repeated using simple age acceleration values defined as residuals from linear regression of clock scores on age and estimated proportion of neurons. (**B**). Multiple-regression residuals for these clocks computed in the test dataset from ROSMAP data were correlated to each other (**C**) and various clinical and pathological scores of AD across samples (**D**).

Multiple epigenetic clocks have previously been implicated as meaningful correlates to AD [24, 26, 40]. We hypothesized that the improved correlations between AD neuropathology and PCBrainAge acceleration are a result of the improved reliability arising from the PC clocks methodology [29]. Therefore, we measured the reliability of 5 previously reported epigenetic clocks in a dataset of 34 cerebellum technical replicates [41]. Reliability, according to the ICC values, is highest in PCHorvath1 and PCBrainAge, the two clocks trained according to the PC Clocks framework (Figure 5A). Age acceleration, defined as the residual of regression of clock values onto age and an estimate of the proportion of neurons, is also most reliable for PCHorvath1 and PCBrainAge (Figure 5B).

To illustrate the proposed effects of increased reliability, these same clocks were then calculated in the ROSMAP test dataset of 700 individuals. Age acceleration values were defined as the residuals of regressing clock scores onto age and proportion of neurons (as well as sex in the case of PhenoAge, for which it is also a significant covariate). These clock residuals were compared to each other (Figure 5C), as well as with previously discussed measures of AD neuropathology, clinical AD diagnosis, and APOE ε4 carrier status (Figure 5D), using Pearson’s correlation values. While PCBrainAge and PCHorvath share a similar degree of reliability, PCBrainAge shows much higher correlation to indicators of AD and AD risk. Further, while PCBrainAge and DNAmCortical together show similar correlations, the increased correlation of PCBrainAge acceleration with AD indicators may arise from its reduced noise and higher reliability as seen in technical replicates.

### Alzheimer’s pathology correlates with PCBrainAge across multiple brain regions

Aging may have distinct effects on different brain regions with respect to atrophy, dendritic morphology, synaptic plasticity, and vasculature [42]. This may be reflected in epigenetic clocks, which indicate measurable differences in brain aging rates between regions [43]. The typical progression of AD involves the reproducible, staged invasion of neurofibrillary tangles [28] and amyloid-β aggregations [27] through the brain. Though AD progression can be variable, some regions show amyloid or tau pathology earlier than others [44]. It is unknown whether regional differences in epigenetic age might help explain the differential impact of AD pathology amongst brain regions.

**Figure 6:**
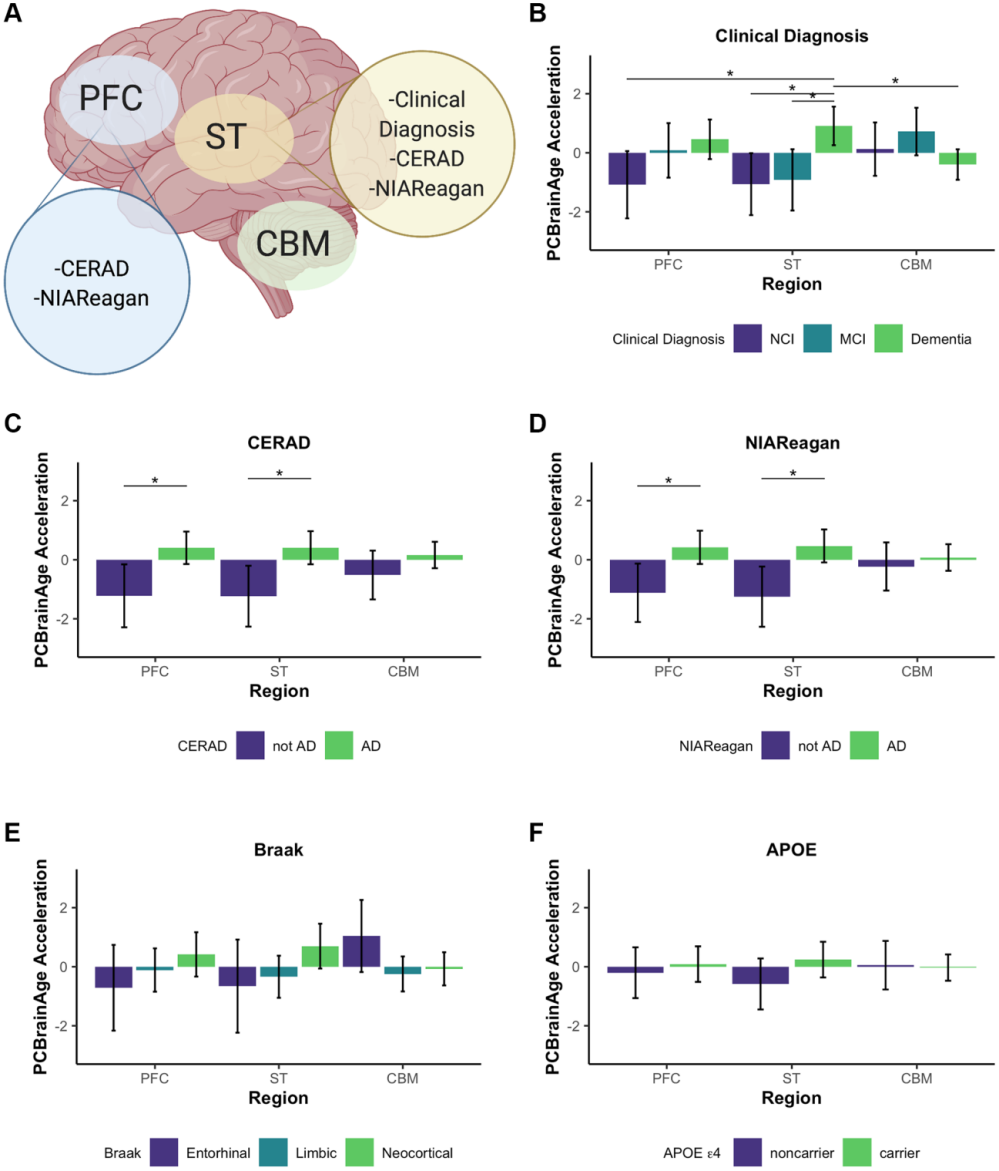
**Multi-region methylation data recapitulates strong PCBrainAge acceleration associations in test data.** Conclusions drawn from significant differences in PCBrainAge are graphically outlined by brain region, created with BioRender.com (**A**). Barplots show the mean PCBrainAge Acceleration as defined by the residual of our mixed linear effects model (eq. 1), with error bars corresponding to a 95% confidence interval. (^*^) denotes Benjamini Hochberg corrected *p*-values < 0.05, where within-region significant comparisons are predominantly highlighted. Acceleration was compared among brain regions between groupings according to clinical diagnosis (**B**), CERAD scores (**C**), NIA-Reagan scores (**D**), Braak Scores (**E**), and APOE ε4 carrier status (**F**).

We used PCBrainAge to measure the aging trends across multiple brain regions and evaluate region-specific associations with AD. Using 333 individuals’ samples from an APOE ε4 carrier enriched subcohort of ROSMAP (Supplementary Table 1), we generated novel DNAm data from 3 distinct brain regions for each individual: Prefrontal cortex (PFC), Striatum (ST), and Cerebellum (CBM) (Figure 6A). This incorporated 212 overlapped individuals for which DNAm data for DLPFC (a distinct region and tissue slice) was available in the original test dataset used here. As done for the original test dataset, principal components were projected into this data, followed by PCBrainAge prediction for each independent region and sample. To account for repeated measurements and to improve modeling of epigenetic age acceleration, we employed a linear mixed effects (LME) model to utilize data across regions in tandem. The model is described in equation 1. The three brain regions tested here are expected to diverge in their epigenetic age prediction based upon data in prior clocks [45–47]. Therefore, we allowed a random effect to the model intercept with age according to brain region. Comparison of this model and simple regression is shown in Supplementary Table 4.


$$\begin{array}{cc} PCBrainAge=Age+PropNeurons+(1|Region) & \left(\text{Eq}\text{. 1}\right) \end{array}$$

We then related age acceleration in each region to AD neuropathology, clinical and pathologic diagnoses, and APOE ε4 carrier status. To account for multiple comparisons, we used a *p*-value adjustment according to a Benjamini Hochberg procedure from a Kruskal Wallis test of nonparametric mean differences (Table 2). Age acceleration in prefrontal cortex and striatum were both associated with premortem clinical diagnosis (Figure 6B), CERAD score (Figure 6C) and NIA-Reagan neuropathological criterion (Figure 6D), though not with Braak scores (Figure 6E). While age acceleration in the striatum trends higher in accordance with APOE ε4 carrier status, there was no significant difference in PCBrainAge acceleration as seen in the larger test dataset (Figure 6F). It is important to note that the dataset used here is a subset of the test dataset used in Figure 3 (See Supplementary Figure 4). The associations here are weaker compared to Figure 3 likely due to the reduced power, as this dataset contains fewer samples. This effect is magnified when comparing multiple brain region samples for each individual which increases complexity of comparisons without an increase in the number of individuals.

**Table 2:** Multi-region PCBrainAge acceleration’s correlates to AD.

	**All**	**PFC**	**ST**	**CBM**
**CERAD**	1.1E-04^*^	6.3E-03^*^	6.3E-03^*^	0.13
**Braak**	0.087	0.25	0.067	0.15
**NIA-Reagan**	4.5E-04^*^	6.3E-03^*^	6.3E-03^*^	0.36
**Clinical Diagnosis**	6.3E-03^*^	6.3E-03^*^	4.7E-03^*^	0.13
**APOE**	0.24	0.52	0.076	0.68

^*^Indicates a *p*-value < 0.05 of Kruskal Wallis nonparametric tests of means across criterion groups, followed by adjustment according to the Benjamini Hochberg procedure when pooling all *p*-values in the table.

The cerebellum has long been characterized as relatively spared in AD, though this has been challenged recently [46]. Interestingly, the cerebellum ages slowly according to the multiple epigenetic clocks, and existing epigenetic clocks do not show correlations between cerebellum age acceleration and AD neuropathology [45] (Supplementary Figure 5). These other clocks were not trained in brain tissue, so it remains plausible that brain—or even cerebellum specific—epigenetic aging signatures are correlated with AD. However, we found that PCBrainAge acceleration in cerebellum is not significantly correlated with AD diagnosis, neuropathology, or APOE ε4 carrier status (Figure 6). Thus, PCBrainAge validates prior reports that the cerebellum’s methylation age diverges from that of other brain regions reflecting its distinctive biology in AD.

Taken together, PCBrainAge demonstrates associations with AD neuropathology, diagnosis, and APOE ε4 carrier status in three regions affected by AD (DLPFC, PFC, and striatum) but not in a region that may be relatively spared in AD (cerebellum). Furthermore, PCBrainAge is applicable in multiple brain regions despite being trained specifically in DLPFC.

**Figure 7:**
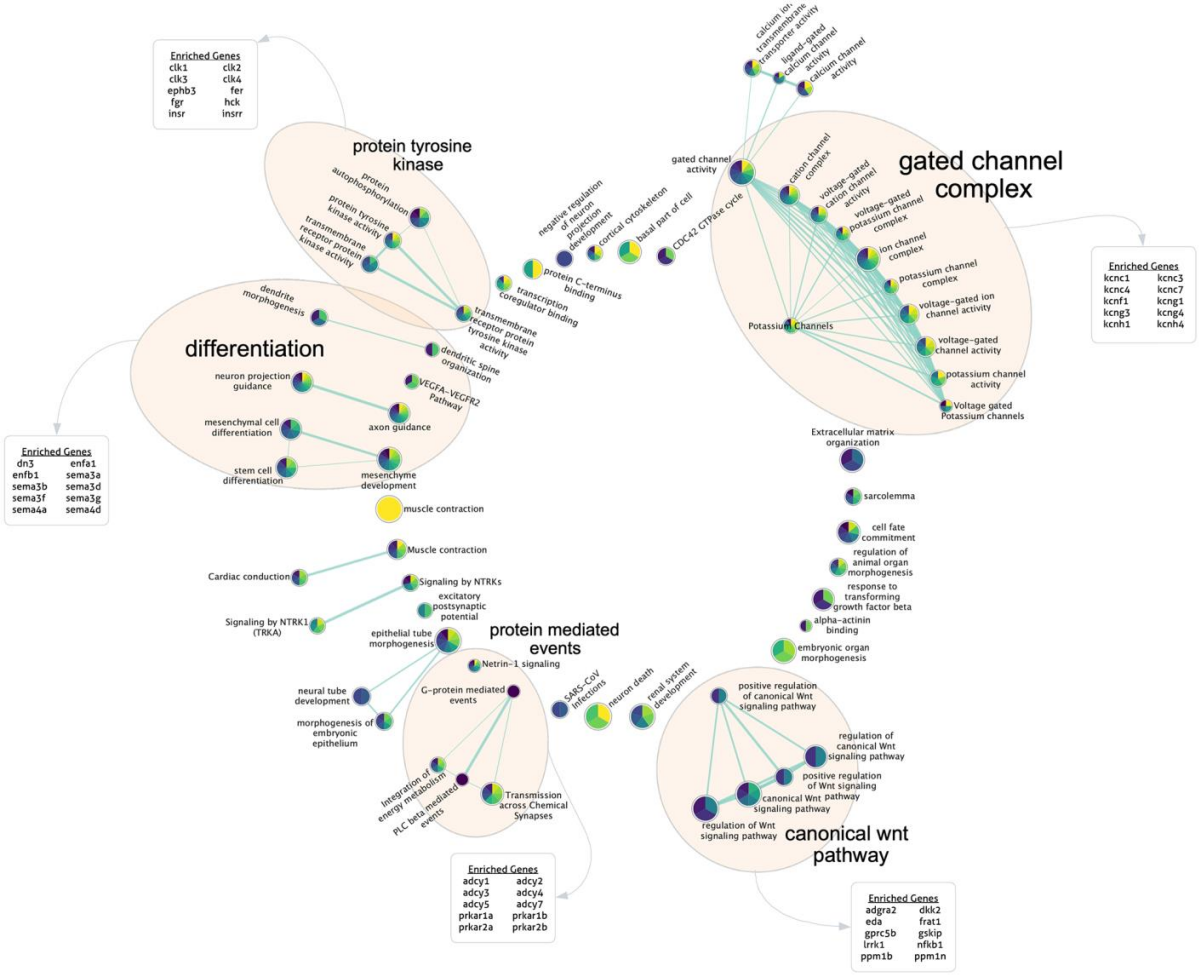
**Gene set enrichment analysis for highly contributing CpGs.** Each PC’s ranked CpG weights were translated to genes according to annotations, and pathway enrichment analysis was run for each PC. Here, the sparse consensus network of enriched curated GO and REAC terms across the 15 PCs is visualized. Annotated clusters of significant pathway similarities and high weights are labeled (5), along with the genes enriched within that group beyond the rest of the network. Each node is colored according to the enrichment score of that term, from PC1 (yellow) to PC15 (dark purple) according to the viridis color palette, with more color slices demonstrating enrichment across more PCs.

Finally, to gain further insight into potentially important biological mechanisms impacting PCBrainAge scores, we performed a modified gene set enrichment analysis. While most previously reported DNA methylation clocks produce a weighted average of a sparse set of CpGs, here we report a metric relying upon a sparse set of principal components which each represents a patterning of weights across all CpGs in the training set. Therefore, we used a ranked gene set enrichment analysis (see methods for further details) using each PC’s CpG absolute loading scores, multiplied by the standard deviation of those PCs in the ROSMAP DLPFC test dataset. This methodology allowed us to approximate the contribution of each CpG to each PC, providing a ranked order list. Once mapped to genes, we generated 15 independent gene set enrichment lists (one for each PC) according to curated GO terms and REAC terms, following a standard protocol [48]. These were used to create a consensus enrichment map with imposed sparsity to encourage formation of a network with similarity across PCs for ease of interpretability in the context of the overall PCBrainAge predictor (Figure 7).

We found 5 major clusters of enriched terms of note, which support PCBrainAge as a brain age predictor and provide further insight into potential underlying mechanisms: The canonical Wnt pathway; protein mediated events; and protein tyrosine kinase. A further two categories are supportive of PCBrainAge’s brain tissue specificity: differentiation; and gated channel complex. These categories were outlined according to network connectivity and structure, and their labels were further verified according to enrichment of top 10 genes within group nodes against all network nodes.

## DISCUSSION

While epigenetic clocks trained in blood, or multiple tissues, can reflect age in brain tissue [49], biomarkers trained specifically for the brain may more accurately capture its aging trends. Clocks trained in peripheral tissue can reflect postmortem AD pathology when applied to brain DNAm data [40]. However, with the exception of the PhenoAge clock [24], the acceleration captured does not typically demonstrate significant association with AD dementia status, despite clear correlations with neuropathologically mediated cognitive decline [40]. This may reflect the intermediate complexity between molecular pathological change and higher order cognitive changes [50]. However, blood or pan-tissue trained clocks may not adequately capture brain aging, due to the brain’s unique methylation profile [51–53], extreme diversity of specialized neuronal [54] and glial [55] cell types, and distinct developmental patterns [56]. In fact, over-reliance upon blood and multi-tissue clocks is likely to ignore unconserved and tissue-specific DNA methylation aging signature [38, 57–60]. Thus, a methylation-based predictor of age in the brain is useful for studying age-related patterns of change in neurodegenerative disease at its source.

Prior work has been done to develop methylation-based predictors of age in the brain in humans [25] and mice [61]. A biomarker of human brain aging, DNAmClock_Cortical_, addresses the systematic underestimation of age in older adults when predicting brain age by existing clocks. While DNAmClock_cortical_ can achieve near-perfect age correlation in brain tissue, this was not the goal of the present model. The lower correlation of PCBrainAge in the test datasets, as depicted in Figure 3, carries important biological signal. While the present clock does not achieve the degree of correlation of clock age and sample age at death found by DNAmClockcortical, PCBrainAge’s utility lies in the robust link between an individual’s PCBrainAge residual (age acceleration), and pathological characteristics of AD. Beyond this, generation of PCBrainAge employed a novel methodology that allowed few samples for adequate training and was shown to reduce technical noise, thereby improving confidence in biological interpretation of the reported age residuals. Reduction of technical noise in this manner has also been hypothesized to reduce the sample size needed to train a robust epigenetic clock model [29], addressing the marked scarcity of brain tissue in comparison to blood DNAm. In an independent dataset, we find that PCBrainAge logically follows these hypotheses, showing improved reliability over multiple clock counterparts. Furthermore, PCBrainAge demonstrates applicability across brain regions.

Pathway and gene set enrichment analysis indicate that PCBrainAge reflects tissue maintenance, metabolic regulation, neurogenesis, and ion channel activity. These identified pathway groups are supported by prior literature on age associated brain changes. The Wnt pathway has been repeatedly associated with aging. In the aging brain, downregulation of Wnt signaling may relate to the deterioration of healthy stem cell niche [62], and dysregulation of adult neurogenesis [63, 64]. Furthermore, key players in the Wnt pathway, such as DKK1 are implicated in the amyloid and tau pathologies of Alzheimer’s Disease [65]. The Wnt pathway enrichment is not unique to PCBrainAge and has been implicated in other epigenetic clock and DNAm studies [66, 67], perhaps representing the core tissue-independent signal captured by PCBrainAge. Due to the number of Wnt pathway interacting proteins, it is likely that our other identified categories are in part identified along with Wnt pathway enrichment. However, they also have been directly implicated in brain aging phenotypes and neurodegeneration. A recent proteomic analysis also showed differential abundance of protein tyrosine kinases in the ROSMAP brain study between APOE ε4 carriers and non-carriers, and implicated these as key candidates for molecular intervention in incipient AD [68]. Gated ion channels are of clear physiological relevance to the brain—however, recent evidence further suggests that reduced neural activation may protect the aging brain [69]. The five major categories of pathway enrichment we have identified suggests that PCBrainAge’s score is heavily derived from methylation changes in genes involved in metabolic activities, particularly those of neurons. With many enrichment terms being related to ion channels directly, metabolic changes, and tissue maintenance, it is clear that PCBrainAge is well-positioned for the study of brain aging and neurodegenerative disease.

While a connection between DNA methylation and AD neuropathology has been a previously discussed possibility [50, 70], the current work also demonstrates a connection between patterns of DNA methylation change and higher order changes like those to cognition, and significant genetic differences like APOE ε4 status. The acceleration predicted by PCBrainAge is correlated with clinical AD dementia, and pathologic AD, outperforms sex-specific and pooled sex models in both males and females, and can be used across multiple cortical and noncortical brain regions. PCBrainAge is also significantly associated with APOE ε4 status (see Figure 3F), which has not been previously shown with existing blood-based clocks. With APOE ε4 carriers exhibiting PCBrainAge acceleration over their non-carrier counterparts, PCBrainAge is consistent with observations that this genotype significantly increases risk in an age-related manner [71]. PCBrainAge can also detect the interaction between APOE status and cognitive diagnoses, given that APOE carriers show acceleration regardless of diagnosis, while noncarriers with AD show distinct acceleration versus those who appear cognitively normal. One limitation, however, is that our dataset shows an enrichment of APOE ε4 carriers with MCI and dementia over cognitively normal counterparts. Regardless, this may reflect APOE ε4 carriers’ increased neuropathological burden [72, 73], while suggesting that APOE carriers may not be aggressively predisposed to higher order cognitive changes.

PCBrainAge can predict age across multiple brain regions while also capturing heterogeneity relevant to AD in that region. The degree of correlation recapitulates previously described differences in the rate of aging of brain samples [43, 45]. DLPFC, PFC, and ST are routinely impacted by AD pathology, unlike cerebellum [74–76]. We found that PCBrainAge acceleration is associated with AD pathology and dementia status in these regions. This signal is slightly more robust in ST, where age correlation is stronger and separation between pathological groups is more distinct. It has been well characterized that tau and amyloid impact brain regions at varying times and to varying degrees. Further investigation is necessary as to whether a model of epigenetic brain aging reflects a relationship between pathological temporality and epigenetic alterations.

In the cerebellum, PCBrainAge recapitulates aging deceleration reported in previous studies [45]. Here we also show that age acceleration of cerebellum lacks correlation with Alzheimer’s pathology and disease status. The slower predicted rate of aging in CBM conforms to expectations that CBM aging and its relationship to AD are drastically different from other brain regions. Without knowing the causal direction for the link between age related 5mC changes and AD pathology, the mechanisms for this relationship remain unclear. There is some evidence that amyloid beta can reduce methyltransferase activity resulting in global hypomethylation, and cerebellum is relatively spared of amyloid pathology until very late in the disease [77]. Future studies should investigate these mechanisms.

PCBrainAge is a promising predictor of regional brain aging, with demonstrated recapitulation of known aging trends in multiple brain regions. However, beyond tracking the relative aging of various brain regions, it can assess meaningful age-acceleration, or pathological aging. This pathological age acceleration is further correlated to AD neuropathology, clinical AD diagnosis, and APOE ε4 carrier status. PCBrainAge may aid in future investigations linking heterogeneity in the aging process to AD risk and individual resilience.

While PCBrainAge is a useful tool alone, we anticipate that deeper characterization of the biological signal it captures will be made possible when used in tandem with multi-omics data. Specifically, use of RNA sequencing data as another means to track changes in cell composition may elucidate the degree to which PCBrainAge’s cell proportion influenced signal capturing disease-associated shifts in cell proportion, beyond simple age-related changes. Proteomic data in these samples can further highlight the functional changes in cellular activity seen across brain regions. Further, while PCBrainAge uses linear methods (PCA and elastic net linear regression) for training, future work should incorporate nonlinear deep learning methods. This will allow clearer contributions by nonlinear aging signals that may provide significant, but currently less apparent, information about AD-associated aging changes.

## METHODS

### Selection of available DNA methylation data

DNA methylation data was acquired from multiple sources (Supplementary Table 1). The training data was accessed from the Gene Expression Omnibus (GSE74193) [31] as the age range was much wider than in the Alzheimer’s Cohort studied: This has the important effect of increasing the ratio of the range of the variable of interest (age) versus the signal (DNAm) noise due to technical error, biological heterogeneity, and the effect of diseases. All sample methylation β values were generated from the dorsolateral prefrontal cortex (DLPFC) using the Infinium HumanMethylation450K Beadchip (Illumina, San Diego CA, USA) and were used as collected, normalized, and reported by the original authors [31]. Samples under the age of 20 from the original GEO dataset were excluded as it has been shown that development typically has a different aging regime when considering epigenetic clocks [23, 78].

For assessment of PCBrainAge in the context of neurodegeneration and AD, we used the previously collected synapse dataset (syn5850422) [36] which generated Illumina 450K methylation data in postmortem DLPFC of participants in the Religious Orders Study and the Rush Memory and Aging Project (ROSMAP) [79]. Methylation β values were used as originally collected, normalized, and reported by the original authors [80]. Samples were excluded if their clinical diagnosis value was a non-AD primary cause of dementia [81]. Use of these samples would introduce significant uncertainty beyond the scope of the current work. Clinical diagnoses were dementia, mild cognitive impairment, and no cognitive impairment, and Alzheimer’s dementia proximate to death (*n* = 700) [82]. Neuropathologic data included CERAD, Braak, and pathologic AD by NIA-Reagan [83]. AD neuropathology was previously generated for this dataset: Neuritic and diffuse plaques, and neurofibrillary tangles were estimated using count data from silver stain; PHFtau tangle density and β-Amyloid load were each estimated using molecularly specific immunohistochemistry [84].

To estimate reliability of PCBrainAge and other DNAm clocks in the brain, we identified a publicly available dataset containing a cohort cerebellum technical replicates (GSE43414) [41]. In brief, two cohorts—1Ai and 1Aii—contained cerebellum 450k methylation data in 91 samples, and 36 samples of rescanned cerebellum 450k methylation respectively. Samples from the 1Ai and 1Aii cohorts following the authors’ own *dasen* normalization were extracted, with a total of 34 paired test-retest replicates remaining (2 rescan samples were removed due to ambiguous labelling leading to no match between 1Ai and 1Aii). Mean imputation across samples was used to remove missing methylation β values.

### Generation of multi-region brain methylation data

Novel collection of multi-region brain 5′-cytosine DNA methylation data was performed for the current work. This data was collected from frozen brain tissue samples obtained from Rush University’s Religious Orders Study and Rush Memory and Aging Project (ROSMAP) [79]. Frozen tissue was isolated in 349 individuals across three brain regions: Brodmann Areas 10 (prefrontal cortex), 22 (striatum), and cerebellum.

Bulk genomic DNA was extracted from each tissue sample using the Chemagic DNA Tissue100 H24 prefilling VD1208504.che protocol (Perkin Elmer Ref# CMG-1207). In brief, tissue was lysed overnight at 56 degrees in 1 mL Chemagic Lysis buffer and 50 uL Proteinase K. Samples were treated with 80 uL of RNASE A @ 4 mg/uL (AmericanBio Ref# AB12023-00100) for 10 minutes at 56C. Lysis was then transferred to a deep well plate and the extraction performed via the Perkin Elmer Chemagic 360 extraction instrument. Samples were centrifuged at 13000RPM for 1 minute, placed on a magnet and transferred to final 1.5 mL Eppendorf tubes. 25–50 mg of extracted DNA per sample was then used according to the manufacturer’s protocol on the Illumina Methylation EPIC array at the Yale Center for Genome Analysis (YCGA) with sample randomization on each array to mitigate batch effects.

The raw.idat files of bisulfite-converted single-CpG resolution of methylation were processed to obtain β values through ratios of probe intensities, according to standard methods. Using the R ‘minfi’ package [85], Noob normalization was performed on β values. For more information, the method used herein was derived from a prior publication [86]. The raw (syn23633756) and normalized beta value (syn23633757) data have been deposited to Synapse (Sage Bionetworks, Seattle, WA, USA).

The sample phenotype data was provided as previously generated by the Rush University Alzheimer’s Disease Center, and in accordance with prior publications: Individuals’ clinical diagnoses [82] and neuropathologic data [83] were annotated as in the test dataset.

### CpG selection

5′-cytosine DNA methylation was collected on two different arrays across datasets: The 450K and EPIC arrays. Therefore, DNAm was limited to only the intersection of sites between the two arrays. Further, CpGs located on sex chromosomes, as indicated in the Illumina 450K array manifest were excluded. This resulted in retention of 357,852 CpGs.

### Model training

Singular vector decomposition was implemented using the *prcomp* function in the R stats package (v3.6.1). Detailed methods for training and principal component projection can be found in the methods of Higgins-Chen et al. [29]. In brief, centered principal component scores for individuals, understood as an individual’s score based upon their CpG β values undergoing a rotation according to the rotation matrix, are used as inputs for an elastic net regression to predict age at death. That is, rather than using the original beta values for a given individual’s CpGs, the left singular vectors (PCs) are used instead (excluding only the last PC). Elastic net regression was performed using the *glmnet* package in R, according to a mixing parameter (α) of 0.5, utilizing equal parts LASSO and ridge regression with 10-fold cross validation. All fit models were estimated using penalization (λ) corresponding to predicted minimal error. Training of the age predictor was performed thus in an unsupervised manner, and in a dataset without clinical AD individuals. The final PCBrainAge model, which constitutes the “core” model entailed retraining an elastic net model such that PCs were zeroed out if not one of the 15 core principal components which had some nonzero weight in the original three models.

### Estimation of neuron proportion

Along with methylation data, publicly available dataset annotations provided sorted cell proportion estimates, or estimates of neuron and glia proportion calculated using the methylation-based Cell Epigenotype Specific Model (CETS) for R package [87]. In the novel, multi-region dataset, the CETS package was used to estimate the proportion of neurons in each brain region sample.

### Statistical measures

All reported scatterplot and predictor of age at death correlations (and corresponding *p*-values) are the result of correlation tests between means according to the Pearson’s product moment coefficient, presuming standard normal distributions. This is implemented using the R function *cor.test* from the stats package. Correlations with annotations of phenotype, as in Figure 2A, 2B are the result of implementing a biweight midcorrelation, a median-based comparison test that improves sensitivity to outliers. This was implemented using the *bicor* function of the WGCNA package in R.

The *p*-value reported for all barplots are the result of a Kruskal-Wallis Rank Sum Test, which is a nonparametric test of means. This did not require assumptions of normality, and was applied using the *kruskal.test* function in the R stats package. Error bars on all barplots represent a standard error of the mean, unless explicitly noted otherwise.

In all tables offering many independent *p*-value comparisons (Table 2, Supplementary Table 2), adjustment of *p*-values were necessary. All adjusted *p*-values were reported following implementation of the Benjamini Hochberg procedure, and values *p* < 0.05 were considered significant.

Reliability of cerebellum replicate data was quantified using a two-way, single-rater, consistency model of intraclass correlation coefficient (ICC) using the *icc* function of the R irr package. This function was used to obtain mean values and asymmetric 95% confidence intervals of ICC for both paired replicate clock values, and clock accelerations as defined by taking a residual upon regressing clock predictions onto age and the CETS-predicted proportion of neurons.

Linear mixed effects (LME) models were used in the context of the multiregion data. This required implementation of the *lmer* function of the lme4 package in R [88]. LMEs were optimized according to a Nelder Mead optimizer, and were visualized using the *sjPlot* package. Generation of LMEs were done in a hypothesis driven manner as described in the results, and were compared to the marginal R^2^ of less complex models. Visualization of the model table was performed using sjPlot [89]. The residuals from the LME model were used to define the age acceleration of each specimen, as used in Figure 6.

### Gene set enrichment analysis (GSEA)

To rank CpGs in order of importance for each PC, CpG loadings were multiplied by standard deviation of the CpG in the ROSMAP DLPFC test cohort [36]. Due to computational limitations of downstream tools, only the top 10% of CpGs thus ranked were used. The ranked list for each PC was then mapped using the Illumina 450k array annotation file to its associated gene, where applicable. Unannotated CpGs were ignored, and duplicate gene entries were consolidated to the first unique instance. A custom background list of CpGs was generated by identifying all unique genes annotated by Illumina for the 450k array.

GSEA proceeded according to a slightly modified version of a standard protocol [48]. Each PC was entered into g:Profiler as a ranked order list of genes, with custom background and Bonferroni *p*-value adjustment. Only non-electronically annotated GO and REAC terms were used for enrichment analysis.

All 15 sets of enrichment terms were used in EnrichmentMap in Cytoscape to generate a consensus network with q-value cutoff of 1 × 10^−3^ and a sparse network to increase agreement across PC enrichment sets. yFiles radial layout was used to visualize the network, AutoAnnotate was used to identify clusters of highly interconnected nodes, and WordCloud was used to generate the top 10 genes overrepresented in each node cluster and verify the labels assigned to each of the 5 groups. Each node is visualized as a pie chart depicting the relative normalized enrichment score of contributing PCs, with PC1 as yellow and PC15 as dark purple following the viridis color palette. More colors within a node demonstrates more conservation of enrichment across PCBrainAge PCs, and an overrepresentation of a slice within a node coloring suggests more contribution to that PC’s own network.

## Supplementary Materials

Supplementary Figures

Supplementary Tables
