# Supplementary material for: Aging the brain: multi-region methylation principal component based clock in the context of Alzheimer’s disease
Source: Aging (Albany NY). 2022 Jul 30;14(14):5641–68. doi: 10.18632/aging.204196 (PMC9365556; doi:10.18632/aging.204196)
Supplement: Supplementary Tables [file aging-14-204196-s002.pdf]

SUPPLEMENTARY TABLES

Supplementary Table 1. Dataset access, annotation and composition.

	Training	Testing	Validation
**Accession**	GSE74193	syn5850422	syn23633757
n	399	700	333
**Array**	450k	450k	EPIC
**Brain Region**	DLPFC	DLPFC	Matched PFC, ST, CBM
**Ages**	20−97	66−108	66−108
**Has AD?**	No	Yes	Yes
**White**	0.48	1	0.97
**Female**	0.34	0.64	0.65
**APOE ε4**	N/A	0.26	0.71

Supplementary Table 2. Biweight midcorrelation of core PCs and AD characteristics.

	Age	*p*	Cerad	*p*	Braak	*p*	NIA-Reagan	*p*	Cogdx	*P*	APOE ε4	*p*
PC1	**−0.10**	*	−0.07		−0.05		−0.04		−0.07		−0.01	
**PC5**	**−0.14**	***	**−0.13**	**	**−0.13**	**	**−0.16**	***	**−0.15**	**	**−0.07**	
PC6	−0.01		−0.05		−0.04		−0.09		−0.07		−0.08	
PC8	0.04		**−0.14**	**	−0.07		−0.06		−0.08		−0.03	
PC9	−0.03		−0.05		−0.04		−0.02		−0.01		−0.07	
PC10	**−0.11**	*	0.05		−0.03		0.00		−0.06		0.06	
PC12	**0.11**	*	−0.07		−0.01		0.01		−0.02		−0.09	
PC15	0.05		0.00		−0.01		0.00		**0.11**	*	−0.03	
PC19	**0.28**	***	0.06		**0.10**	*	0.10	*	0.09		−0.06	
PC26	0.01		0.08		0.01		0.00		0.02		0.00	
PC29	0.00		0.07		0.04		0.02		0.02		0.09	
PC32	**−0.18**	***	−0.02		**−0.12**	*	−0.08		**−0.14**	**	0.03	
**PC33**	**−0.23**	***	**−0.20**	***	**−0.17**	***	**−0.17**	***	**−0.15**	**	0.00	
PC41	**0.09**	*	−0.08		0.01		−0.03		0.02		−0.07	
PC391	0.03		−0.03		0.05		0.02		0.04		0.03	
**Overall**	**0.59**	***	**0.15**	***	**0.28**	***	**0.27**	***	**0.32**	***	**0.03**	
**Acceleration**	−0.03		**0.15**	***	**0.13**	**	**0.17**	***	**0.16**	***	**0.12**	*

BH Corrected *P*-values > 0.05 (n.s.) $^{*}p < 0.05$, $^{**}p < 0.005$, $^{***}p < 0.0005$.

Supplementary Table 3. Linear models for PCBrainAge.C acceleration.

**Predictor**	**Testing**		**PFC**		**ST**		**CBM**	
	**Estimates**	***p***	**Estimates**	***p***	**Estimates**	***p***	**Estimates**	***p***
(Intercept)	33.33	<0.001	54.76	<0.001	48.05	<0.001	47.20	<0.001
Age	0.46	<0.001	0.39	<0.001	0.46	<0.001	0.17	<0.001
Prop N	3.88	0.138	−18.58	<0.001	−16.80	<0.001	−18.94	0.031

Observations	700	333	333	333
R^2/R^2 adjusted	0.389/0.388	0.254/0.250	0.310/0.306	0.085/0.080

Supplementary Table 4. Linear mixed effect models in multi-region brain data improves upon OLS regression.

Predictors	OLS Regression		LME Model	
	**Estimates**	***p***	**Estimates**	***p***
(Intercept)	63.79	<0.001	49.03	<0.001
Age	0.35	<0.001	0.34	<0.001
Prop N	−35.68	0.138	−15.96	<0.001
**Random Effects**				
σ2			18.48	
τ00			169.63_{region}	
ICC			0.90	
N			3_{region}	
**Observations**	997		997	
**R^2/R^2 adjusted**	0.325/0.324		0.030/0.905	
